# Exogenous GA3 Application Can Compensate the Morphogenetic Effects of the GA-Responsive Dwarfing Gene *Rht12* in Bread Wheat

**DOI:** 10.1371/journal.pone.0086431

**Published:** 2014-01-20

**Authors:** Liang Chen, Liugen Hao, Anthony G. Condon, Yin-Gang Hu

**Affiliations:** 1 State Key Laboratory of Crop Stress Biology in Arid Areas and College of Agronomy, Northwest A&F University, Yangling, Shaanxi, China; 2 CSIRO Plant Industry, Canberra, Australia; 3 Institute of Water Saving Agriculture in Arid Regions of China, Northwest A&F University, Yangling, Shaanxi, China; Nanjing Agricultural University, China

## Abstract

The most common dwarfing genes in wheat, *Rht-B1b* and *Rht-D1b*, classified as gibberellin-insensitive (GAI) dwarfing genes due to their reduced response to exogenous GA, have been verified as encoding negative regulators of gibberellin signaling. In contrast, the response of gibberellin-responsive (GAR) dwarfing genes, such as *Rht12*, to exogenous GA is still unclear and the role of them, if any, in GA biosynthesis or signaling is unknown. The responses of *Rht12* to exogenous GA_3_ were investigated on seedling vigour, spike phenological development, plant height and other agronomic traits, using F_2∶3_ and F_3∶4_ lines derived from a cross between Ningchun45 and Karcagi-12 in three experiments. The application of exogenous GA_3_ significantly increased coleoptile length and seedling leaf 1 length and area. While there was no significant difference between the dwarf and the tall lines at the seedling stage in the responsiveness to GA_3_, plant height was significantly increased, by 41 cm (53%) averaged across the three experiments, in the GA_3_-treated *Rht12* dwarf lines. Plant height of the tall lines was not affected significantly by GA_3_ treatment (<10 cm increased). Plant biomass and seed size of the GA_3_-treated dwarf lines was significantly increased compared with untreated dwarf plants while there was no such difference in the tall lines. GA_3_-treated *Rht12* dwarf plants with the dominant *Vrn-B1* developed faster than untreated plants and reached double ridge stage 57 days, 11 days and 50 days earlier and finally flowered earlier by almost 7 days while the GA_3_-treated tall lines flowering only 1–2 days earlier than the untreated tall lines. Thus, it is clear that exogenous GA_3_ can break the masking effect of *Rht12* on *Vrn-B1* and also restore other characters of *Rht12* to normal. It suggested that *Rht12* mutants may be deficient in GA biosynthesis rather than in GA signal transduction like the GA-insensitive dwarfs.

## Introduction

Gibberellins (GAs) are a major class of plant hormones that regulate plant growth and development, from seed germination and stem elongation to fruit-set and growth [1]–[4]. It is important for plants to produce and maintain optimal levels of bioactive GAs to ensure normal growth and development. Mutants with impaired GA biosynthesis or response show typical GA-deficient phenotypes, such as dark green leaves, dwarfism and late-flowering, while elevated exogenous GA dose or increased signaling can cause excessive plant growth and earlier flowering [5]–[8]. Mutants deficient in GA biosynthesis can be rescued by exogenously applied GAs but this is not possible if the mutation is in the GA signaling pathway [7], [8].

The deployment of genes influencing plant height through the GA pathway was a major factor in the success of the Green Revolution, which created high-yielding cultivars of rice and wheat with shorter and sturdier culms [9]. In contrast to the recessive, semi-dwarf *sd-1* Green Revolution allele in rice, which is a loss-of-function mutation in one of the major GA biosynthetic genes (*GA20ox2*) [10]–[12], the reduced height *Rht-B1b* (*Rht1*) and *Rht-D1b* (*Rht2*) Green Revolution alleles in wheat are semi-dominant gain-of-function mutations causing impaired GA signaling and thus conferring dwarfism through constitutive repression of cell division and elongation [13]. The wheat Green Revolution genes are orthologues of the *Arabidopsis* GA-insensitive (*gai*), the rice slender1 (*slr1*) [14] or the rice *gai* [15], the barley slender1 (*sln1*) [16], [17] and the maize dwarf-8 (*d8*) [13] genes. However, in addition to reducing plant stature, *Rht-D1b* and *Rht-B1b* also reduce seedling vigour and coleoptile length, and may reduce crop water-use efficiency [18]–[21] and performance in some unfavorable environments [22]–[24]. So, opportunities exist for replacing *Rht-B1b* and *Rht-D1b* in wheat with alternative dwarfing genes, such as the GA-responsive dwarfing genes (*Rht4*, *Rht5*, *Rht9*, *Rht12*, *Rht13*, *Rht14*, *Rht15, Rht16* or *Rht18*). These genes have been reported to reduce plant height without compromising early plant growth [25]. Even though there are several GA-responsive dwarfing genes in wheat, their molecular characteristics remain obscure [26], [27] and the mechanisms by which they resulted in a reduction of plant height is not well understood. Fortunately, the metabolic pathways of gibberellin biosynthesis, deactivation and signaling have become relatively clear and many of the genes involved have been identified [28], [29], which lays the foundation for analysis of GA-responsive dwarfing genes in wheat.

Generally, dwarfing genes in wheat are classified into two categories, GA-responsive (GAR) and GA-insensitive (GAI), reflecting the relative magnitude of their responses to application of exogenous GAs [25], [30]. GA-responsive dwarfing genes show significantly enhanced growth response to exogenous GAs (probably have mutations in GA biosynthesis pathway) while GA-insensitive dwarfing genes show very little response to exogenous GAs (probably have mutations in GA signaling pathway, such as *Rht-D1b* and *Rht-B1b*) [25]. This classification has usually been conducted at the seedling stage, for example, based on the response of coleoptile length or the first seedling leaf elongation rate to exogenous GAs [25], [30]. There is less information available on the response of the GAR dwarfing genes to exogenous GAs at later growth stages.

*Rht12*, a dominant dwarfing gene from the gamma ray-induced mutant Karcagi 522M7K of winter wheat (here referred to as Karkagi-12), has been classified as a GA-responsive dwarf gene [31]. We have previously reported the effects of *Rth12* on wheat using a F_2∶3_ population derived from a cross between Ningchun 45 (a tall spring wheat cultivar) and Karcagi-12 in two field experiments [32]. *Rht12* significantly decreased stem length (43%∼48% for peduncle) and leaf length (25%∼30% for flag leaf), while the thickness of the internode walls and width of the leaves were increased. Additionally, the *Rht12* dwarf lines showed very dark green leaves compared to tall lines. *Rht12* significantly decreased plant height, by around 40%, while seedling vigour, coleoptile length and root traits at the seedling stage were not affected adversely. *Rht12* lines had significantly increased floret fertility and grain number and achieved a higher harvest index (due to the lower plant biomass) than the tall genotypes. However, *Rht12* extended the duration of the spike development phase, especially the duration from sowing to double ridge, and delayed anthesis date by around 5 days. Even the dominant *Vrn-B1* allele could not compensate for these effects on phenological development, which may hamper the direct utilization of *Rht12* in wheat breeding. Another negative effect of *Rht12* on yield components was that grain size was reduced significantly. Similarly, other studies have found that *Rht12* had a substantial effect on reducing plant height without altering early vigour and significantly increased spikelet fertility, harvest index, and lodging resistance but these were usually accompanied by delayed ear emergence and reduced grain weight. [31], [33]–[35].

Although *Rht12* has been classified as a GA-responsive dwarfing gene [25], [33], a comprehensive understanding on the response of *Rht12* to exogenous GAs is lacking. Thus, the role of *Rht12*, if any, in GA biosynthesis or signaling is still unclear. Moreover, it has been recently found that the effects of the dwarfing gene *Rht8*, which had been considered as ‘GA-responsive’, was possibly not due to defective GA metabolism or signaling because the wild type and *Rht8* lines responded with a very similar increase in final plant height (15% and 13%, respectively; *P*<0.05) with GA_3_ application. It has been proposed that the effects of *Rht8* are possibly due to reduced sensitivity to brassinosteroids [26]. So, can exogenous GA_3_ restore the characters of *Rht12* (such as plant height, flowing time or seed size) to normal? Is there a significant difference between *Rht12* lines and the tall lines in GA_3_-responsiveness?

To date none of the dwarfing genes have been cloned except a few GA-insensitive dwarf genes [27]. More information on these genes is needed for a better understanding of how the GAR dwarf genes act on plant growth and what their roles are in GA biosynthesis or signaling. The aim of this paper was to extend the work of Chen *et al.* [32] by examining the response of *Rht12* to exogenous GA_3_ on plant development, agronomic traits and yield components and to investigate the possible role of *Rht12* in GA biosynthesis or signaling. The response to exogenous GA_3_ of *Rht12* dwarf and tall lines were compared to determine if *Rht12* was responsive to exogenous GA_3_ during the whole life cycle of wheat and if *Rht12* dwarf lines had a notably greater responsiveness to exogenous GA_3_ than tall lines.

## Materials and Methods

### General Description

The experiments were carried out in the experimental field of the Institute of Water Saving Agriculture in Arid Regions of China, Northwest A&F University, Yangling, Shaanxi, China, during two growing seasons of 2011–2012 and 2012–2013. Supplemental irrigation was provided as needed to avoid water stress. Weeds were manually removed where necessary, and fungicides and insecticides were applied to prevent diseases and insect damage. Weather data were recorded at an automated weather station at the site.

### Plant Material

A cross was made using Ningchun 45 as the female and Karcagi-12 as pollen donor in May, 2009 as reported by Chen et al [32]. Karcagi-12 (*Triticum aestivum* L.) is the mutant carrying the dominant GA-responsive dwarfing gene *Rht12* and shows strong winter habit with the recessive loci for all three *Vrn-1* genes. Ningchun 45 (*Triticum aestivum* L.) is a tall Chinese spring wheat cultivar which carries the dominant gene *Vrn-B1* and lacks any known dwarfing genes detectable by molecular markers.

The presence or absence of the loci for *Rht12* and *Vrn-B1* in each F_2_ individual was determined using the corresponding molecular markers (for details see [32]). Individuals within the four groups of homozygous genotypes of *Rht12Rht12Vrn-B1Vrn-B1* (abbreviated as RRBB), *Rht12Rht12vrn-B1vrn-B1* (RRbb), *rht12rht12Vrn-B1Vrn-B1* (rrBB) and *rht12rht12vrn-B1vrn-B1* (rrbb) were then selected and used to develop the F_2∶3_ and, subsequently, F_3∶4_ lines for further analysis.

The two parents and 24 random F_2∶3_ homozygous lines (of a total of 57 homozygous lines identified) were used in the experiments to evaluate the effects of exogenous GA_3_ on *Rht12*. Among the F_2∶3_ homozygous lines there were 7, 7, 5 and 5 lines with the genotypes *RRBB*, *RRbb*, rrBB, and rrbb, respectively. There were two sowing dates in 2011–2012 growing season: October 6, 2011 (Autumn Sowing, AS) and February 6, 2012 (Spring Sowing, SS). In 2012–2013 growing season, the 24 F_3∶4_ lines were sown on October 6, 2012 (Autumn Sowing, AS). The lines and parents were sown in plots of four rows that were 2 m long and 25 cm apart, with seeds spaced 5 cm apart within rows. The parents and the 14 dwarf and 10 tall lines were randomly arranged to avoid competitive effects with two replications.

### Exogenous Gibberellic Acid (GA_3_) Treatments

GA_3_ was applied on the dwarf and the tall genotypes and the two parents. GA_3_ solution (100 µM or 35 mg/L) was sprayed using a small aerosol or smeared on the leaves and culm surface by cotton at several stages of development: the 5 leaf stage (Z15), tillering stage (Z21), stem elongation stage (Z31), early booting stage (Z41), early heading stage (Z51), early anthesis stage (Z61) and early kernel and milk development stage (Z71) [25], [26], [36]. For each plant, 1–2 mL GA_3_ solution was applied at the first 2 developmental stages and 3–5 mL at the later 5 stages. Control plants were treated with the same solution without GA_3_ [26], [37].

### Coleoptile Length and Seedling Root Characters

Coleoptile length was measured from the seed to the tip of the coleoptile with a ruler after germination in a darkened growth chamber irrigated with water or GA_3_ solution (100 µM) at 20°C after 200°Cd using the method described [22], [38]. The characteristics of seedling leaf 1 and seedling root were assessed using a ‘Cigar’ method as described previously [32]. Seedling vigour was evaluated as the area of the first leaf and coleoptile length.

### Spike Development and Fertility

Beginning at the 5 leaf stage (Z15), three plants were randomly taken from each plot every 3 days to observe spike differentiation. The main shoot was dissected to determine the timing of double ridge formation (DR), as described [39] using a digital Stereo Microscope (Nikon, SMZ1500). The timing of heading (Z55) and anthesis (Z65) was visually determined when 50% of the plants in the plot had reached the stages.

At anthesis, five plants were harvested in the central row of each plot, and then the number of fertile florets in the main shoot spike was counted. Florets were considered fertile when the stigmatic branches spread wide, with either pollen grains present on them or with green anthers [40].

### Plant Height, Leaf and Internode Character

Seedling height was measured in 10 plants for each plot as the distance from the soil surface to the ligule of the last fully emerged leaf, and the elongation rate of different lines was analyzed weekly from Z12 (two leaf stage) until heading. Seedling dry weight was calculated from 3 plants of each line every week to determine the plant growth rate. The number of leaves emerging on the main shoot was determined on 5 plants per plot [41]. At maturity, ten random plants for each plot were measured to get the mean plant height as the distance from the soil surface to the top of the ear (awns excluded).

The lengths of internodes (mm) were measured from the mid-point of their subtending nodes. Stem diameter (mm) was taken at the middle of each internode with digital calipers (TESA Etalon). Internodes were then cut at their centre point and the thickness (mm) of stem wall was obtained using digital calipers, with two measurements on opposite sides of the stem, then a mean value was calculated [42]. The characters of the internodes were measured when they fully developed.

At maturity, lodging was scored as the fraction of the area affected and the severity of lodging in those areas noted, on a scale of 0 for a standing crop to 90 for a crop flat on the ground [36].

### Yield Components and Yield

At maturity, the main shoot ears of 10 plants of each line were measured for assessment of spike length, spikelets per spike, grains per spike and fertile shoots per plant. Due to the frequent sampling prior to maturity about 20 to 40 plants remained in each plot for investigating the average above-ground biomass per plant, average yield per plant, harvest index and 1000-grain weight.

### Data Analysis

For each parameter measured, the mean value for each homozygous class (7 RRBB, 7 RRbb, 5 rrBB and 5 rrbb lines) was calculated. Because the previous study [32] found that there was no significant difference between BB and bb genotypes on many traits in either dwarf or tall lines, statistical evaluation of the data was carried out as two categories (14 RR and 10 rr lines) by ANOVA analysis with multiple comparisons (LSD test at the 0.05 level) using the statistical package SPSS18.0. 0°C was chosen as base temperature, whenever thermal time was used to estimate developmental progress.

## Results

### Seedling Vigour

**Figure 1:**
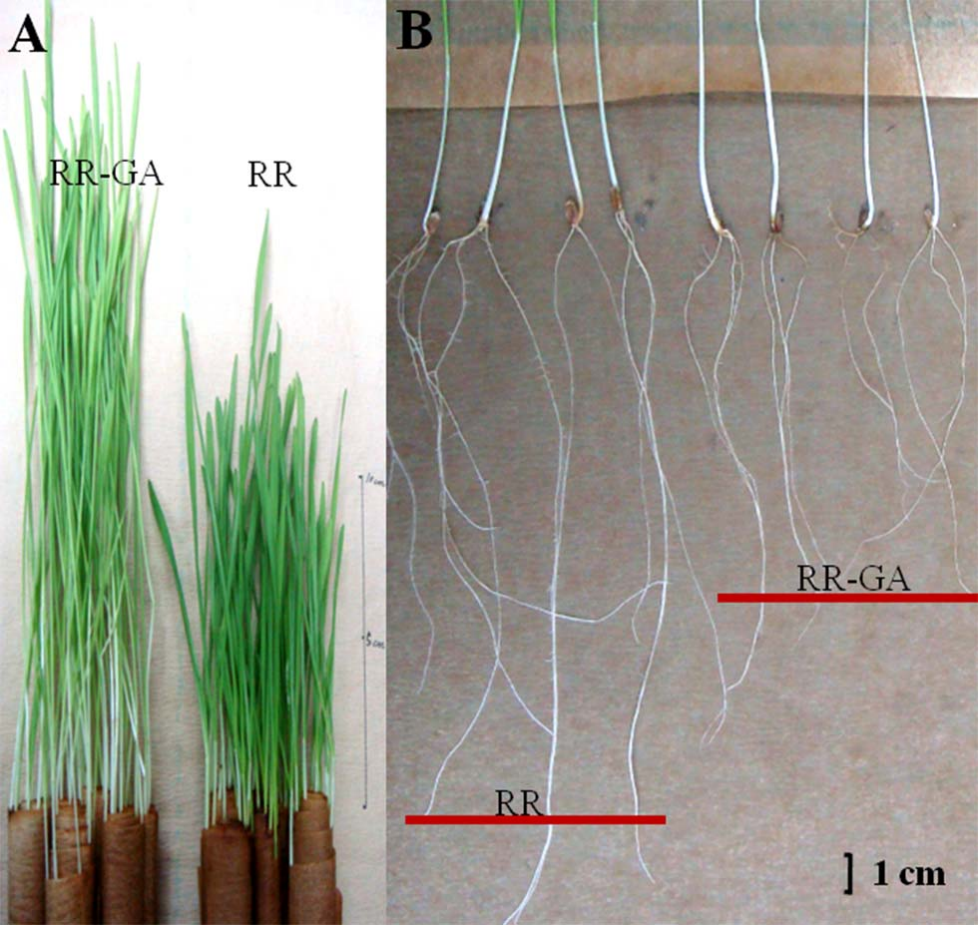
Seedling leaf and seminal root morphology of the *Rht12* dwarf plants with and without GA_3_-treatment. Panel A shows the difference between seedlings of untreated dwarf plants (RR) and GA_3_-treated dwarf plants (RR-GA) growing in rolled filter-paper ‘cigars’. The RR plants had shorter leaf length and dark green leaves while the RR-GA plants had longer leaves and that were lighter green. Panel B shows the difference in the seminal root growth between RR and RR-GA dwarf plants grown in rolled ‘cigars’. GA_3_ significantly reduced root length (and root dry mass) though it promoted above-ground growth in the dwarf lines, suggesting that GA_3_ had negative effects on seedling root growth in the *Rht12* dwarf plants.

In the F_2∶3_ and F_3∶4_ lines, no significant difference was observed for the length or width of seedling leaf 1 between the tall and dwarf groups in the absence of exogenous GA_3_. In the treatments with exogenous GA_3_, the area of seedling leaf 1 significantly increased in both the dwarf and tall lines. The *Rht12* dwarf lines had a small but significant increase in leaf length over the tall lines, however, there was no notable difference in its response to exogenous GA_3_ between dwarf and tall lines (Table 1; Fig. 1). Additionally, coleoptile lengths of the dwarf and tall lines in the GA_3_ treatments were both significantly increased compared to that of the control treatments in both years (29% and 41% in the dwarf lines and 13% and 24% in the tall lines, respectively) (Table 1). There was no significant difference in coleoptile length of *Rht12* genotypes compared with the corresponding tall lines, either with or without additional GA_3_. Summarizing, in comparison with the wild-type tall, *Rht12* had no major effect on the growth of seedling leaf 1 or coleoptile length and also on their responsiveness to exogenous GA_3_.

**Table 1:** Seedling vigour characteristics of F_2∶3_ and F_3∶4_ lines with *Rht12* dwarf and tall genotypes, with and without GA_3_ treatment.

Year	genotype*	Leaf 1 length (cm)	Leaf 1 width (cm)	Leaf 1 area (cm^2^)	Coleoptiles length (cm)
2011–2012	RR	10.0±0.38c	0.50±0.04a	4.0±0.61b	4.9±0.60b
	RR-GA	13.1±0.85a	0.48±0.08b	5.0±0.98a	6.3±0.82a
	rr	10.3±0.55c	0.52±0.05a	4.3±0.64b	5.2±0.49b
	rr-GA	11.9±0.71b	0.50±0.05a	4.8±0.74a	5.9±0.56a
2012–2013	RR	9.8±0.62c	0.46±0.05a	3.6±0.52b	4.6±0.54b
	RR-GA	13.6±0.88a	0.45±0.07a	4.9±0.90a	6.5±0.88a
	rr	9.9±0.57c	0.48±0.04a	3.8±0.58b	5.0±0.55b
	rr-GA	12.5±0.91b	0.47±0.05a	4.7±0.77a	6.2±063a

* RR: dwarf lines with the dominant *Rht12*; RR-GA: dwarf lines with exogenous GA_3_ application; rr: tall lines; rr-GA: tall lines with exogenous GA_3_ application. All data are means ±SD of each group. There was no significant difference in these traits between BB and bb genotypes. Different letters within columns indicate statistically significant differences (*P<*0.05).

Exogenous GA_3_ had negative effects on seminal root growth in both dwarf and tall lines. The total root length and root dry mass of the tall and *Rht12* dwarf lines was significantly decreased in the GA_3_ treatments and the dry mass ratio of root to shoot was also significantly reduced due to the increased shoot dry mass as well (Fig. 1).

### Spike Development

For the three experiments in the two years, spike development was significantly delayed and the duration of the spike development phase was extended in the *Rht12* dwarf lines compared to that of tall lines. There was no significant difference between RRBB and RRbb groups in these effects. Compared to the tall lines with winter growth habit (rrbb), time to double ridge of the *Rht12* dwarf lines either with *Vrn-B1* or with *vrn-B1* was delayed by 16 days (45°Cd), 5 days (89°Cd) and 23 days (105°Cd) in the three experiments respectively (Table 2). Thus, the effect of the dominant vernalization gene *Vrn-B1* was masked in the *Rht12* dwarf lines. In contrast, the tall lines without *Rht12* but with *Vrn-B1* (rrBB) reached double ridge about 64 days (30°Cd), 4 days (57°Cd) and 48 days (55°Cd) earlier than those with *vrn-B1* (rrbb) in the three experiments, respectively, indicating that tall plants having dominant *Vrn-B1* needed less time to undergo vernalization. Application of exogenous GA_3_ significantly affected the progress of spike development and shortened the duration of the pre-anthesis phase in the dwarf lines (Table 2). It especially shortened the time to double ridge. The RRBB-GA lines (*Rht12* dwarf lines with *Vrn-B1*, with GA_3_ application) showed 39 days (18°Cd), 5 days (70°Cd) and 24 days (25°Cd) shorter SW-DR than the RRbb-GA lines (*Rht12* dwarf lines with *vrn-B1*, with GA_3_ application) in the three experiments, respectively. It seemed that exogenous GA_3_ relieved the epistatic effects of *Rht12* on *Vrn-B1* in the dwarf lines, such that RRBB-GA plants displayed a spring-like phenotype while RRbb-GA still showed a winter phenotype. Moreover, tall lines with GA_3_ treatment also reached double ridge quicker, by about 2–4 days than the untreated tall plants, though this effect was not as significant as in the *Rht12* dwarf lines (Table 2). The flowering of *Rht12* dwarf lines was delayed by about 5–8 days compared to tall lines while there was no significant difference between BB and bb lines in either year (Table 2). However, the GA_3_-treated *Rht12* dwarf lines flowered earlier by almost 7 days compared to those without GA_3_ treatment, while the GA_3_-treated tall lines flowered only 1–2 days earlier than the tall lines without GA_3_ treatment. It was therefore clear that GA_3_ had a greater effect on spike development in the *Rht12* dwarf genotypes than in the tall genotypes. Application of GA_3_ broke the masking effect of *Rht12* on *Vrn-B1*, indicating either that *Rht12* lines had serious defects in the GA metabolic pathway or that exogenous GA_3_ could in some other way compensate for the negative effects on plant development by *Rht12*.

**Table 2:** Spike phenological development of the four genotypic combinations with and without GA_3_ treatment in the three experiments.

Genotype	Autumn-sown 2011–2012				Spring-sown 2011–2012				Autumn-sown 2012–2013			
	SW-DR	DR-AN	SW-AN	Total leafNo.	SW-DR	DR-AN	SW-AN	Total leafNo.	SW-DR	DR-AN	SW-AN	Total leaf No.
RRBB	153.0(702.2)a	61.0(853.6)a	214.0(1555.8)a	14.1a	74.0(566.0)a	40.0(760.5)a	114.0(1367.5)a	11.0a	146.0(779.1)a	61(854.8)	207.0(1633.9)a	14.0a
RRbb	154.0(705.0)a	61.5(879.6)a	215.5(1584.6)a	14.2a	75.0(582.1)a	40.0(800.0)a	115.0(1385.1)a	10.9a	146.5(782.0)a	62.5(882.1)	209.0(1664.1)a	14.0a
rrBB	73.0(629.0)e	136.0(808.7)b	209.0(1437.7)b	12.4c	65.0(428.0)e	43.0(803.6)a	108.0(1231.6)b	9.2c	75.0(620.8)e	126(895.9)a	201.0(1516.7)b	12.0c
rrbb	137.0(658.2)b	73.5(810.2)b	210.5(1468.4)b	13.5b	69.0(485.3)b	40.0(771.3)a	109.0(1256.6)b	10.1b	123.0(675.9)b	79.5(869.5)	202.5(1545.4)b	13.0b
RRBB-GA	96.0(639.3)d	111.0(766.7)c	207.0(1406.5)d	12.5c	63.0(400.2)d	45.0(831.4)b	108.0(1231.6)b	10.5b	96.5(642.1)d	103.5(857.9)	200.0(1500.0)b	12.0c
RRbb-GA	135.0(657.6)b	72.0(748.9)c	207.0(1406.5)d	12.4c	68.0(470.3)b	41.0(786.3)a	109.0(1256.6)b	10.5b	120.0(667.2)c	81.5(859.8)	201.5(1527.0)b	12.0c
rrBB-GA	71.0(626.3)e	137.0(795.3)b	208.0(1421.6)c	12.2c	62.0(382.6)d	46.0(849.0)b	108.0(1231.6)b	9.1c	73.0(619.6)e	127(880.4)a	200.0(1500.0)b	12.0c
rrbb-GA	133.0(655.1)c	75.0(766.5)c	208.0(1421.6)c	13.5b	67.0(453.4)c	41.0(778.2)a	108.0(1231.6)b	10.0b	120.5(668.5)c	80.0(838.5)	200.5(1507.0)b	12.5b

Data are the duration days (d) with calculated thermal time (°C d) in parenthesis; SW: sowing date, DR: double ridge formation date, AN: anthesis date. All data are means of each genotype. Statistical analysis was carried out using the thermal time. Different letters within columns indicate statistically significant differences (*P<*0.05).

Due to a longer duration to double ridge, *Rht12* dwarf lines developed more leaves than the equivalent tall genotypes. However, due to the shorter SW-DR of the GA_3_-treated *Rht12* dwarf plants, less leaves were produced in the GA_3_-treated *Rht12* dwarf plants than that in the untreated ones (Table 2). Despite this, the number of elongated internodes was not changed. In this study, the dwarf parent Karcagi-12 generated the largest number of leaves in all experiments due to its longest duration of SW-DR while the GA_3_-treated Karcagi-12 had almost 4 leaves less than the untreated ones.

### Plant Height and Associated Traits

**Figure 2:**
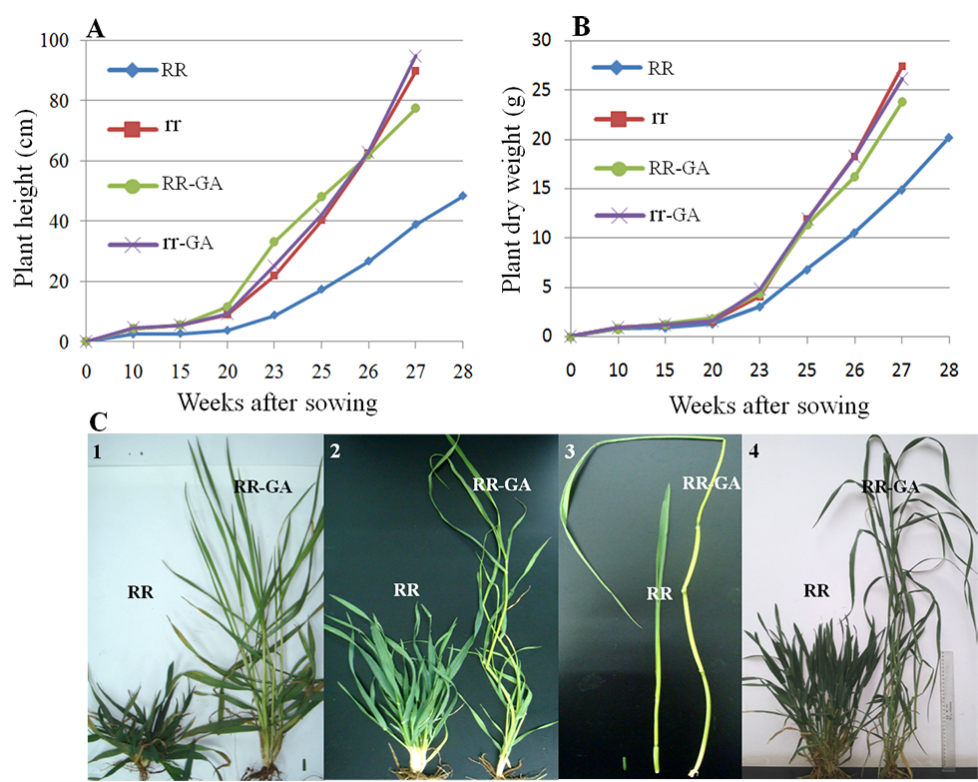
Development of plant height from the soil surface to the top ligule (A), the dynamic changes of plant dry weight from sowing to heading (B) and the phenotypic appearance (C) of *Rht12* dwarf lines and tall lines under GA_3_ treatments in the 2012–2013 growing season. A: development of plant height of the RR, RR-GA, rr and rr-GA plants. The final height was achieved at week 28 for RR plants but at week 27 for rr, rr-GA and RR-GA plants. B: the dynamic changes of plant dry weight in the plants. C_1_: RR and RR-GA plants taken from the field at the 20^th^ week; C_2_: RR and RR-GA plants at the 23^th^ week; C_3_: the main stem of RR and RR-GA plants at the 23^th^ week. The RR-GA plants had produced three internodes while the RR plants only elongated one or two internodes; C_4_: RR and RR-GA plants at the 26^th^ week. RR: *Rht12* dwarf lines; rr: tall lines; RR-GA: *Rht12* dwarf lines with GA_3_ application; rr-GA: tall lines with GA_3_ application.

**Figure 3:**
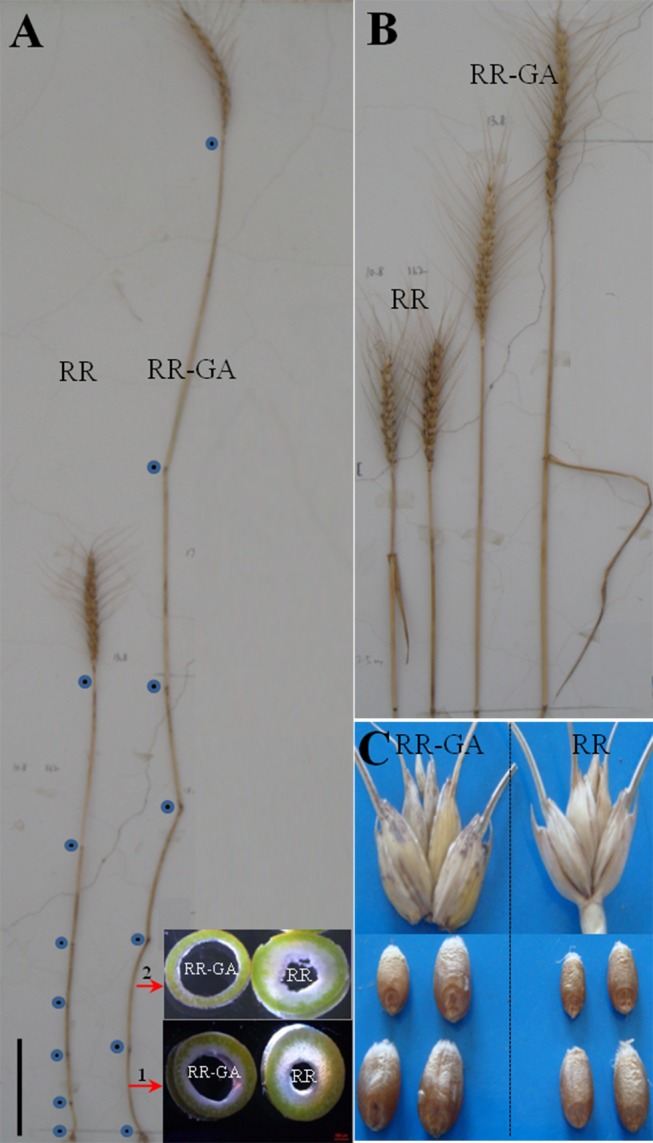
Phenotypic effects on internode length and wall thickness, spike and seed size in the 2012–2013 growing season for plants of *Rht12* dwarf lines with (RR-GA) and without (RR) GA_3_ treatment. A: the main culm morphology of RR and RR-GA plants. The wall thickness of the first and the second internode at the base of the stem are shown at the bottom right. The blue dots indicate the nodes of the main culm. Scale bar  = 10 cm. B: spike length of the RR and RR-GA lines. RR-GA plants had longer spikes as well as a longer peduncle and flag leaf than RR plants (*P*<0.05). C: spikelet morphology and seed size of the RR and RR-GA plants. The RR-GA plants had bigger seed than RR plants (*P*<0.05).

**Figure 4:**
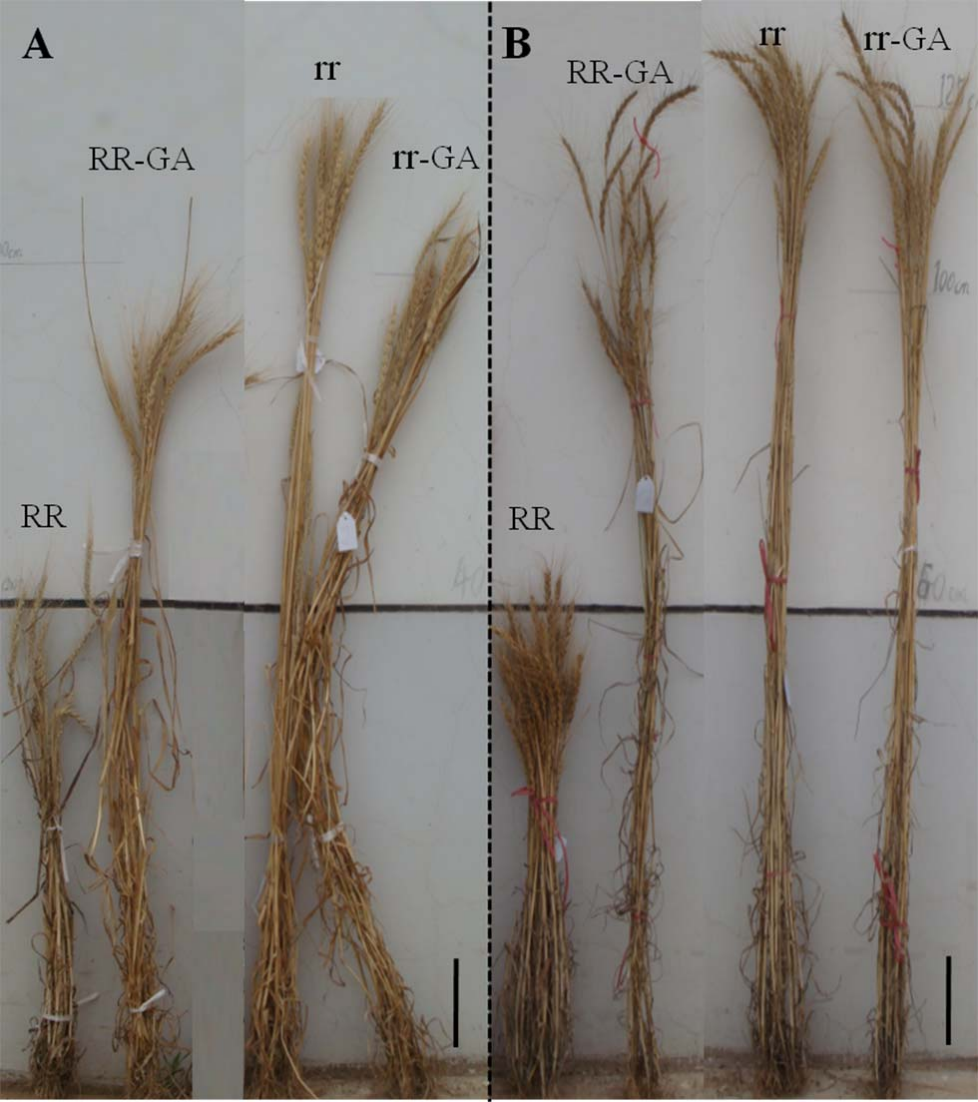
Gross plant morphology of *Rht12* dwarf (RR) and tall (rr) lines and of GA_3_-treated (RR-GA; rr-GA) lines in the AS experiments of 2011–2012 and 2012–2013 growing seasons. A: mature plant morphology of the two categories in the AS experiment of 2011–2012 growing season. The stems of the GA_3_ treated plants are bent because of serious lodging. B: mature plant morphology of the two categories in the AS experiment for the 2012–2013 growing season. The rr-GA lines were generally <10 cm taller than rr lines while RR-GA lines had a significant increase in plant height. Scale bar  = 10 cm.

The culm elongated faster in tall lines than in *Rht12* dwarf lines from seedling stage, with the tall lines reaching jointing stage earlier and producing longer internodes and more biomass than the *Rht12* dwarf lines (Fig. 2). This difference was sustained to maturity in both years. However, *Rht12* dwarf lines achieved a higher resistance to lodging through shorter internode length and increased wall thickness without altering internode diameter compared with the tall lines. In particular, the lengths of the first and second internodes at the base of the stem, was significantly decreased in the *Rht12* dwarf lines while their wall thickness was increased by 0.24 mm (31%) and 0.22 mm (34%) in the AS experiment, 0.23 mm (24%) and 0.23 mm (29%) in the SS experiment in the 2011–2012 growing season and 0.13 mm (15%) and 0.17 mm (22%) in the 2012–2013 growing season, respectively (Table 3). These shorter and thicker internodes might confer a greater resistance to lodging in the dwarf plants. However, after exogenous GA_3_ application, the *Rht12* dwarf plants showed a faster stem elongation rate, similar to that of tall plants, compared with the untreated *Rht12* dwarf plants (Fig. 2). The lengths of the first and second internodes at the base of the stem were significantly increased in the GA_3_-treated *Rht12* dwarf plants compared with the untreated dwarf plants: by 4.1 cm (117%) and 6.9 cm (115%) in the AS experiment, 6.3 cm (252%) and 7.1 cm (131%) in the SS experiment of the 2011–2012 growing season and 4.3 cm (165%) and 4.8 cm (87%) in the 2012–2013 growing season, respectively. Also, the diameter and wall thickness of those internodes were significantly reduced in the GA_3_-treated *Rht12* dwarf lines and thus these now ‘tall’ GA_3_ dwarf plants had serious lodging, as did the true tall lines (Table 3;Fig. 3A). Treating *Rht12* dwarf plants with exogenous GA_3_ recovered the lost ability to produce long internodes and achieved a final plant height similar to that of the tall lines. Compared with the untreated *Rht12* dwarf plants, plant height was increased by 40 cm (49%), 45 cm (62%) and 37 cm (48%) in the GA_3_-treated ones in the AS and SS experiments of 2011–2012 and AS experiment of 2012–2013, respectively (Table 3 and Fig. 4). In contrast, the internode characters and plant height of the tall lines were not affected significantly by GA_3_ treatment (∼20% and ∼10% increase in the length of the first and the second internode at the base of the stem, less than 10 cm (<10%) increase in plant height), suggesting that the tall plants were not as sensitive as the *Rht12* dwarf plants to exogenous GA_3_ due to the possible difference in the GA metabolic pathways of the two height categories or the tall lines were likely saturated at endogenous levels of GA. Despite this increase in response to application of endogenous GA, the plant height of the RR-GA lines remained lower than those of the tall lines.

**Table 3:** Length, diameter and wall thickness of the first and second internode at the base of the stem of *Rht12* dwarf and tall genotypes with and without GA_3_ treatment in the three experiments.

Year and Expt	Genotype	1^st^ internode			2^nd^ internode			Plant height(cm)	Lodgingscore
		LT (cm)	DI (mm)	WT (mm)	LT (cm)	DI (mm)	WT (mm)		
2011–2012-AS	RR	3.5±0.66d	4.11±0.25a	1.02±0.10a	6.0±0.65d	4.17±0.17a	0.87±0.11a	81.3±7.25d	0.0
	RR-GA	7.6±1.92a	2.95±0.21b	0.57±0.08c	12.9±2.01a	3.12±0.14b	0.43±0.08c	121.5±19.07c	45.0
	rr	4.8±0.86b	4.13±0.24a	0.78±0.10b	10.2±0.63c	4.21±0.18a	0.65±0.09b	127.4±14.77b	40.0
	rr-GA	5.6±1.09c	4.10±0.25a	0.80±0.10b	11.1±0.80b	4.18±0.18a	0.62±0.09b	133.0±16.54a	40.0
2011–2012-SS	RR	2.5±0.85d	3.75±0.20a	1.18±0.11a	5.4±0.97d	3.91±0.20a	1.03±0.09a	73.7±4.28d	0.0
	RR-GA	8.8±2.66a	3.06±0.18b	0.71±0.06c	12.5±3.10a	3.10±0.18b	0.63±0.08c	119.5±20.14c	10.0
	rr	3.9±1.08c	3.78±0.16a	0.94±0.08b	8.6±1.05c	3.90±0.22a	0.80±0.10b	124.2±13.65b	8.5
	rr-GA	5.0±2.13b	3.72±0.16a	0.88±0.07b	9.8±1.85b	3.88±0.21a	0.77±0.11b	132.6±16.02a	8.0
2012–2013-AS	RR	2.6±0.70d	4.07±0.24b	1.01±0.10a	5.5±0.83d	4.15±0.19a	0.94±0.11a	77.6±6.74d	0.0
	RR-GA	6.9±2.40a	3.05±0.23c	0.64±0.07c	10.3±2.08a	3.13±0.17b	0.50±0.09c	114.5±18.83c	60.0
	rr	4.5±0.93c	4.15±0.22a	0.88±0.10b	9.9±1.47c	4.20±0.20a	0.77±0.12b	125.5±12.60b	60.0
	rr-GA	5.4±1.20b	4.13±0.23a	0.86±0.10b	10.7±1.62b	4.23±0.20a	0.75±0.11b	129.7±14.08a	65.0

Note: AS: autumn-sown experiments; SS: spring-sown experiments. LT is internode length; DI is internode diameter at the mid-point; WT is wall thickness. All data are means ±SD of each genotype. Different letters within columns indicate statistically significant differences (*P*<0.05).

### Spike Characters and Floret Fertility

Spike length, number of spikelets per spike and number of florets initiated per spike were all decreased by *Rht12* compared with the tall lines. However, *Rht12* significantly increased the number of fertile florets per spike and achieved a higher fertility than the tall lines (Table 4). If the *Rht12* dwarf lines could maintain higher fertility and increase the number of florets initiated per spike after applying exogenous GA_3_, more grains would be produced resulting in increased yield. Unfortunately, in the AS experiments of both years, exogenous GA_3_ increased spike length and also number of spikelets per spike in the *Rht12* dwarf lines (Fig. 3B) but the number of florets initiated per spike and fertile florets per spike were significantly decreased and the fertility was reduced compared with untreated dwarf ones (Table 4). This lower fertility might have been due to there being less dry matter partitioned to spikes to support floret survival during the late reproductive phase as more available assimilates were transported to the larger vegetative organs in the now ‘tall’ GA_3_-treated *Rht12* dwarf plants. There was no significant difference on spike characters between the GA_3_-treated and untreated plants in the true tall lines though the number of florets initiated per spike was slightly reduced in the GA_3_-treated tall plants.

**Table 4:** Spike characters and fertility of *Rht12* dwarf and tall genotypes with and without GA_3_ treatment in the three experiments.

Year and Expt	Genotype	Spike length (cm)	Spikelets spike^−1^	Florets initiated spike^−1^	Fertile florets spike^−1^	Fertility*
2011–2012-AS	RR	13.6±1.14c	20.1±0.94b	196.2±16.60a	49.1±3.56a	0.25±0.01a
	RR-GA	15.2±1.43a	20.5±1.06a	174.4±20.81c	40.3±5.04d	0.23±0.02b
	rr	14.3±0.98b	20.6±1.17a	200.5±15.44a	44.5±3.68b	0.22±0.01b
	rr-GA	14.5±1.02b	20.4±1.14a	192.0±18.62b	43.1±4.47c	0.22±0.01b
2011–2012-SS	RR	14.1±0.98c	20.7±1.24b	200.3±18.66c	38.7±1.98d	0.19±0.01b
	RR-GA	15.8±1.55a	21.0±1.38b	190.6±22.40d	44.0±2.65c	0.23±0.02a
	rr	15.1±0.84b	21.5±1.07a	213.4±19.53a	50.1±2.34a	0.23±0.01a
	rr-GA	15.4±0.83b	21.5±1.10a	205.3±20.47b	48.0±2.47b	0.23±0.01a
2012–2013-AS	RR	13.4±0.76b	20.5±1.01b	199.2±18.45b	49.7±3.55a	0.25±0.01a
	RR-GA	14.9±1.30a	21.0±1.22a	184.2±21.39c	43.0±4.26c	0.23±0.01b
	rr	14.5±0.77a	21.1±0.97a	211.0±17.79a	45.6±3.99b	0.22±0.01b
	rr-GA	14.7±0.81a	21.2±1.28a	206.3±20.37b	45.1±4.21b	0.22±0.01b

* , Fertility is estimated as the ratio of fertile florets to total floret number per spike of the main shoot spike. All data are means ±SD of each genotype. Different letters within columns indicate statistically significant differences (*P*<0.05).

Possibly due to slower development of the vegetative phase (SW-DR), which shortened the period of time available under favorable conditions prior to flowering, the *Rht12* dwarf lines produced fewer (23%) fertile florets per spike than the tall lines in the SS experiment (see [32]). Exogenous GA_3_ accelerated plant development in dwarf lines and provided more time under favorable conditions to produce fertile florets in the *Rht12* dwarf plants. The fertility of GA_3_-treated *Rht12* dwarf lines was 21% higher than that of the untreated ones in the SS experiment (Table 4).

### Yield and Yield Components

For the AS experiments in both years, grain numbers per spike were significantly increased whereas 1000-grain weight and plant biomass were significantly decreased in *Rht12* dwarf lines compared to the tall lines. The increased grain numbers and the larger number of fertile ears per plant of the dwarf lines resulted in there being no significant difference in plant yield between the dwarf and tall lines. Additionally, since plant biomass was significantly decreased in the *Rht12* dwarf lines, there was a net increase in harvest index (Table 5). Exogenous GA_3_ increased plant height of *Rht12* dwarf lines as well as plant biomass but it reduced the number of fertile florets, which resulted in a lower grain number per spike and lower plant yield (Table 5). This was despite exogenous GA_3_ increasing seed size in the *Rht12* dwarf lines (Fig. 3C). The 1000-grain weight of the GA_3_-treated *Rht12* dwarf plants was significantly increased, by 9.2 g (28%), 3.5 g (12%) and 6.5 g (18%), compared with untreated dwarf plants in the three experiments, respectively (Table 5). This indicated that GA_3_ could partially compensate for the substantial negative effect of *Rht12* on yield components, although the increased 1000-grain weight of the GA_3_-treated *Rht12* dwarf lines was still less than that of the tall lines. Additionally, the number of efficient spikes per plant and plant yield were decreased in the GA_3_-treated *Rht12* dwarf lines compared with the untreated ones. Thus the harvest index of the GA_3_-treated *Rht12* dwarf lines was significantly reduced, by 25% and 18% in the two AS experiments, compared with that of the untreated plants.

**Table 5:** Yield components and harvest index of *Rht12* dwarf and tall genotypes with and without GA_3_ treatment in the three experiments.

Year and Expt	Genotype	Grain number spike^−1^	1000-grainweight (g)	Number of efficient spikes plant^−1^	Plant yield* (g)	Plant biomass* (g)	Harvest index
2011–2012-AS	RR	45.6±3.87a	32.3±3.24c	15.3±1.32a	12.7±2.34b	35.5±6.50c	0.36±0.02a
	RR-GA	39.0±4.47d	41.5±4.71b	12.2±1.89c	10.8±2.94c	39.6±7.05b	0.27±0.05c
	rr	41.5±3.62b	43.2±4.40a	13.0±2.01b	13.2±2.52a	42.5±6.72a	0.31±0.03b
	rr-GA	40.2±3.98c	43.4±4.47a	12.1±2.33c	12.6±2.70b	42.0±6.80a	0.30±0.04b
2011–2012-SS	RR	35.7±2.63d	30.4±3.15c	2.5±0.44c	3.7±0.43c	12.1±2.47c	0.31±0.01a
	RR-GA	42.1±3.91c	33.9±4.06b	3.4±0.70b	4.5±0.90b	19.0±2.29b	0.24±0.04c
	rr	47.1±3.74a	36.5±3.66a	3.8±0.73a	5.5±1.26a	20.0±4.62a	0.28±0.03b
	rr-GA	45.9±3.83b	36.2±3.61a	4.0±0.81a	5.3±1.10a	19.7±4.50a	0.27±0.03b
2012–2013-AS	RR	47.7±4.02a	37.0±3.52c	13.2±2.11a	14.0±2.14b	36.7±6.07c	0.38±0.02a
	RR-GA	42.0±4.93d	43.5±4.68b	10.5±2.56c	13.1±2.73c	41.9±7.14b	0.31±0.04c
	rr	43.5±3.75b	44.7±4.30a	11.5±1.64b	14.6±2.01a	44.5±6.85a	0.33±0.02b
	rr-GA	42.6±3.90c	44.2±4.57a	10.8±1.88c	14.0±2.17b	44.0±6.88a	0.32±0.02b

* , These data are the mean values of 20∼40 plants per plot. All data are means ±SD of each genotype. Different letters within columns indicate statistically significant differences (*P*<0.05).

In the SS experiment, the *Rht12* dwarf lines performed poorly for yield components due to their slow development rate. Grain number, 1000-grain weight, tiller number, plant biomass and plant yield of the *Rht12* dwarf lines were all lower than for the tall lines. GA_3_-treated *Rht12* dwarf lines had a faster development rate and flowered earlier than the untreated ones, which contributed to achieving higher plant yield. Grain number, seed size, tiller number, plant biomass and plant yield were all increased in the GA_3_-treated *Rht12* dwarf lines compared with the untreated ones. Harvest index of the GA_3_-treated *Rht12* dwarf lines was reduced due to the significantly increased plant height and biomass (Table 5).

In all three experiments in the two years, plant biomass and height and 1000-grain weight of the GA_3_-treated *Rht12* dwarf lines were all increased compared with the untreated ones. These traits of *Rht12* dwarf lines showed a consistent response to exogenous GA_3_ application such that they performed like tall genotypes, indicating that the shorter plant stature and smaller seed size caused by *Rht12* was likely to be a result of a deficiency of endogenous GAs.

Actually, GA_3_ treatment like the dosage here were harmful to the tall genotype (which probably with sufficient GAs). Their endogenous hormone metabolic balance might be disturbed by the excess exogenous GA_3_ application. As observed in this study, some variant genotypes were also founded in the GA_3_ experiment, such as curling flag leaf, abnormal node shape and “closed-flowered” with stamens inside the hard glumes of variants. Some yield components of the GA_3_ treated tall lines performed poorly than that of the untreated tall ones. For example, grain number, number of efficient spikes per plant and plant yield all reduced in the GA_3_ treated tall lines though this decrease was not significant as that in the GA_3_ treated *Rht12* dwarf lines, whereas seed size was not changed significantly in the GA_3_ treated tall lines compared with the untreated tall ones (Table 5). GA_3_ application resulted in lower yield for tall lines. This was associated with fewer grains per spike and fewer fertile spikes per plant. Plant biomass and HI were unchanged by application of GA_3_ to tall lines.

### Effects of Exogenous GA_3_ on the Two Parents Karcagi-12 and Ningchun45

Exogenous GA_3_ had similar effects on the dwarf parent Karcagi-12 as on the dwarf F_2∶3_ and F_3∶4_ genotypes described above. Plant height, plant biomass and seed size were significantly increased by GA_3_ application while harvest index was decreased. In particular, plant height was increased by 70% compared with the untreated plants (Table 6). Plants of GA_3_-treated Karcagi-12 reached double ridge stage earlier than untreated plants, by 25 days (90°Cd), 14 days (222°Cd) and 32 days (200°Cd) in the three experiments, respectively. However, exogenous GA_3_ had smaller effects on the tall parent Ningchun45 compared with Karcagi-12 (Table 6). This difference between the two parents suggested that Karcagi-12 may need additional GAs to promote its development and to obtain a ‘normal’ phenotype and that defects probably exist in GA metabolic pathways in Karcagi-12.

**Table 6:** Duration of pre-anthesis developmental phases and yield components in the two parents with and without GA_3_ application.

Year and Expt	Genotype	SW-DR*	SW-AN*	Plant height (cm)	No. of fertile spikes plant^−1^	Grain number spike^−1^	1000-grain weight (g)	Plant yield (g)	Plantbiomass(g)	Harvestindex
2011–2012-AS	K	162.0(747.1)a	220.0(1661.6)a	77.5±4.69d	15.2±2.32a	43.0±3.99b	29.5±2.75c	12.1±2.13b	33.6±7.67c	0.36±0.02a
	K-GA	137(658.2)b	208.0(1421.6)b	130.7±9.88a	11.8±2.01b	38.2±4.64c	40.1±3.95b	11.0±3.02c	38.9±8.66b	0.28±0.03c
	Nch	65.0(615.7)c	210.0(1458.9)c	122.8±5.58c	10.4±1.28c	45.4±4.34a	50.3±3.02a	13.5±2.01a	40.8±8.50a	0.33±0.03b
	Nch -GA	65.0(615.7)c	210.0(1458.9)c	125.5±7.12b	10.0±1.67c	45.5±5.07a	49.3±3.20a	13.1±2.27a	40.5±820a	0.32±0.03b
2011–2012-SS	K	79.0(650.4)a	121.0(1471.5)a	60.7±3.29d	1.9±0.23c	31.0±1.78c	28.0±3.25c	1.9±0.46c	8.4±0.89c	0.23±0.01b
	K-GA	65.0(428.0)b	110.0(1275.8)b	105.8±6.64c	3.2±0.50b	40.0±4.04b	33.4±4.01b	3.0±0.88b	18.6±2.29b	0.16±0.02c
	Nch	64.0(413.5)c	106.0(1192.8)c	119.0±5.93b	4.3±0.27a	50.1±4.26a	37.2±3.16a	6.3±1.59a	21.5±2.13a	0.29±0.02a
	Nch-GA	63.0(400.2)c	106.0(1192.8)c	123.2±8.55a	4.5±0.48a	49.0±4.22a	37.5±3.07a	6.5±1.74a	22.0±2.30a	0.30±0.02a
2012–2013-AS	K	155.0(875.5)a	214.0(1750.0)a	70.6±4.40b	11.3±2.54a	47.0±4.40b	33.5±3.26c	13.6±2.36b	35.0±6.01c	0.39±0.03a
	K-GA	123.0(675.9)b	202.5(1545.4)b	120.0±7.99a	10.4±2.33b	43.5±5.58c	40.4±4.07b	12.3±2.87c	40.0±7.67b	0.31±0.04c
	Nch	71(615.5)c	200(1500.0)c	120.5±6.60a	8.0±1.26c	48.5±3.97a	51.0±3.58a	15.4±2.21a	42.2±6.52a	0.36±0.02b
	Nch-GA	70(613.6)c	200(1500.0)c	122.7±8.44a	7.5±1.37d	48.0±4.70a	51.2±4.11a	15.0±2.60a	41.7±6.30a	0.36±0.03b

Note: K: the dwarf parent Karcagi-12; Nch: the tall parent Ningchun45. *Data are the duration days (d) with calculated thermal time (°C d) in parenthesis; SW: sowing date, DR: double ridge formation date, AN: anthesis date. Data are means ±SD. Different letters within columns indicate statistically significant differences (*P*<0.05). Statistical analysis of duration performed using thermal time.

## Discussion

This study is part of a series of experiments carried out to obtain a better understanding of the GA-responsive dwarfing gene *Rht12*. In the previous studies, the effects of *Rht12* on plant development and agronomic traits were analyzed comprehensively [32]–[35]. However, a clear understanding of the response of *Rht12* to exogenous GA_3_ is lacking. Moreover, the role, if any, of *Rht12* in GA biosynthesis or signalling or both remains unknown. Here, contrasting homozygous lines with or without *Rht12* and *Vrn-B1* genes (RRBB, RRbb, rrBB and rrbb) were selected in a F_2_ segregating population and assessed as F_2∶3_ and F_3∶4_ lines to evaluate the response of *Rht12* to exogenous GA_3_.

Previous studies showed that *Rht12* had no negative effects on seedling vigour [34], [43]. Here we observed that there was no significant difference between dwarf and tall lines in area of the first seedling leaf or coleoptile length, in response to exogenous GA_3_. A similar result was reported by Ellis *et al.* (2004) who found that the maximum first leaf elongation rate (LER_max_) of *Rht12* dwarf lines and their response to GA was not significantly different to the corresponding tall lines though the dwarf lines had a small but significant increase in LER_max_ (∼23%) over the tall lines (∼37%) in the presence of GA. However, there was no notable difference on seedling vigour between the dwarf and the tall lines in either the presence or absence of GA. Thus, *Rht12* was classified as a late-acting dwarfing gene [25]. Additionally, the GA-insensitive dwarfing genes, such as *Rht-B1b* and *Rht-D1b*, which are characterized by a lack of GA response and verified to be involved in GA signaling, cause a significant reduction in seedling vigour [13], [19], [25]. Moreover, another class of dwarfing genes (which includes *Rht16* and *Rht18*) had reduced seedling vigour compared with the corresponding tall lines while their relative GA response was significantly greater (50∼80%) than the tall lines [25]. It was predicted that these *Rht* mutants were deficient in GA biosynthesis because the reduction in leaf elongation rate could (at least partially) be reversed by the application of GA [25]. Quantifying GA and its precursors in these mutants would test this hypothesis and could pinpoint the biochemical block leading to the reduction in GA. In fact, GA-responsive dwarf mutants identified in many species often result from mutations in genes encoding GA biosynthetic enzymes [1]. In particular, the major semi-dwarfing gene in rice (*sd-1*) has been shown to result from a deficiency in a late step of GA biosynthesis [12].

Even though *Rht12* had no effect on coleoptile length, the area of seedling leaf 1 or responsiveness to exogenous GA at the very early seedling stage, it was found that *Rht12* had a great effect on spike development, tillering, leaf and stem length (especially flag leaf length and peduncle length), harvest index and many other agronomic traits [25], [32]. In this study, the application of exogenous GA_3_ to *Rht12* dwarf lines significantly shortened the duration to double ridge and promoted earlier flowing while reducing the number of emerged leaves. In particular, exogenous GA_3_ broke the masking effect of *Rht12* on *Vrn-B1* such that the RRBB lines recovered to a nearly spring phenotype. In contrast, the tall lines had a much weaker response to exogenous GA_3_ (∼3% for both rrBB and rrbb lines) than the dwarf lines (∼35% for RRBB lines and ∼12% for RRbb lines) on the duration to double ridge. It seemed that additional GA was necessary to ‘assist’ *Vrn-B1* promote spike development in the *Rht12* dwarf lines. It is not known whether any other development-promoting genes, like *Vrn-A1*, *Ppd-D1* or *Ppd-B1*, can compensate for the delay in spike development in *Rht12* lines. In addition to effects on phenological development, *Rht12* decreased internode length and final plant height (∼40%) in the absence of exogenous GA. However, the dwarf lines had a much greater response to the addition of GA_3_ than the tall lines with an increase in plant height by about 50% while this was only 5% in the tall lines. It was clear that the major effect of *Rht12* on spike development and stem elongation could be overcome by GA treatment. This raises the possibility that the *Rht12* mutant is deficient in GA, at least late in development.

*Rht12* has been found to have significantly reduced grain size compared with other wheat dwarf genes [44], [45]. The reason for this is still not clear [32]. It may simply be due to the delayed flowering of *Rht12* lines into a more stressful period. Alternatively, the possible endogenous GA deficiency of the *Rht12* lines might be the main reason for producing smaller seeds. In this study, exogenous GA_3_ significantly increased grain size, although this may have been because seed-set was decreased by GA_3_ treatment. Wang et al (2001) found that the seed-set of the third floret was significantly reduced by GA_3_ application though it did not affect seed-set of the first and second florets [46]. This may not have been a direct effect of GA_3_ application. Poor seed set at the third floret with GA_3_ application may have been due to increased competition for assimilates from the strongly elongating stem. Lower grain number of the GA-treated lines might also contribute to the larger grain size [47], although there are many GA effects that could enhance grain size. For instance: GA application elongated both the lemma and palea of the florets especially in the third and fourth florets in the spikelet [46] and may increase cell length in the pericarp [48]; the increased size of vegetative organs may provide more assimilates available for producing large grains; induced early flowering may extend the period of time available with favorable conditions prior to harvest for grain development [33]. However, the reduction in grain size of different dwarf genes may be through different modes of action from each other [18], [34], [45] and further studies are needed to determine the possible relation between *Rht* genes and grain size as well as GA and grain size.

Even though wheat dwarf genes have been studied for many years, only the GA-insensitive dwarf genes *Rht-B1b* and *Rht-D1b* have been cloned [27]. However, there is still limited information on GA-responsive dwarf genes. *Rht12* is located on chromosome 5AL, linked to Xgwm291 by 5.4 cM. High-resolution mapping should be initiated for eventual map-based cloning of *Rht12*. Alternatively, due to the possibility that *Rht12* is involved in GA metabolic pathways, it would be instructive to use the quantitative RT-PCR strategy to investigate the expression patterns of the known GA metabolism genes (such as *TaKAO*, *Ta20ox*, *Ta13ox*, *Ta3ox* and *Ta2ox*) to look for potential candidate genes for *Rht12*. If the expression of a gene is significantly decreased or even non-expressed in the dwarf plant compared with that of the corresponding tall lines (transcript abundance probably correlated with plant height), this gene could be a candidate gene for sequence analysis. After alignment of sequences of the dwarf plant and the wild type or tall plants, the mutation site may be found in the gene. Indeed, there has been more information collected on genes involved in GA signal transduction than in GA biosynthesis related to wheat *Rht* genes [13], [27], [49], [50], though there have been many successful examples of GA biosynthesis variations correlated with dwarf phenotypes in Arabidopsis and rice [7], [11], [12], [51]. Thus, more information of the genes involved in GA biosynthesis in wheat is needed for a better understand of how these genes act on plant growth and result in dwarfism. On the other hand, the manipulation of GA biosynthesis or perception may be a good target for regulating crop height [49], [52]. However, it is clear that analysis of the endogenous GAs in *Rht12* mutant is a priority.

In conclusion, the *Rht12* dwarf lines grown in this study showed compact and dark green leaves, dwarfism and a late-flowering phenotype compared with the tall lines. All of these are typical features of GA-deficient mutants [8], [52], [53]. Moreover, these effects could be rescued by exogenous GA_3_ treatment, suggesting that this possible defect/mutation was not involved in GA signal transduction like the GA-insensitive dwarfs, but probably in the GA biosynthesis pathway [15], [25], [53], [54]. This study also confirms that reduced plant height could be dissociated from any effects on the early stage of growth [25]. *Rht12* caused a strong form of dwarfism and yet had no effect on coleoptiles length or seedling leaf growth. This type of late-acting dwarf gene in wheat therefore offers the opportunity to reduce plant stature without compromising on early growth and crop establishment [25], [55]. Since *Rht12* lines posses this feature, it is probable that they do not have a deficiency in GA at the early seedling stage. Why this changes later in development is an interesting question.
